# Fulminant Hepatic Failure after Chemosaturation with Percutaneous Hepatic Perfusion and Nivolumab in a Patient with Metastatic Uveal Melanoma

**DOI:** 10.1155/2021/8870334

**Published:** 2021-03-29

**Authors:** Lindsey Teal, Jeffrey Yorio

**Affiliations:** ^1^The University of Texas at Austin Dell Medical School, Austin, Texas, USA; ^2^Texas Oncology, Austin, Texas, USA

## Abstract

Immune checkpoint inhibitors, such as nivolumab, a programmed death receptor-1 (PD-1) inhibitor, have dramatically improved the treatment of advanced melanomas. Chemosaturation with percutaneous hepatic perfusion (PHP) delivers chemotherapy in high doses directly to the liver and is a potentially effective treatment modality in metastatic uveal melanoma with liver metastases. Its safety and effectiveness have not been studied in patients also receiving immunotherapy. A 46-year-old male with a history of uveal melanoma of the right eye was found to have liver metastases. He was treated with PHP using high-dose melphalan for 6 months with a partial response followed by progression. Two months after his last PHP treatment, the patient was started on nivolumab. After two doses of nivolumab, the patient developed severe hepatitis that progressed to fulminant hepatic failure and death despite treatment with high-dose corticosteroids and mycophenolate mofetil. Nivolumab and other immune checkpoint inhibitors have been effective in treating advanced melanoma and extending life. However, there are serious immune adverse events that can occur. While hepatitis after taking nivolumab has been documented, fulminant hepatic failure is rare. We believe that prior PHP treatment contributed to the severity of the hepatitis and, ultimately, fulminant hepatic failure. To our knowledge, this is the only case of fulminant hepatic failure secondary to a checkpoint inhibitor with preceding PHP. Specific precautions should be made in patients who have been exposed to PHP in the past, and further studies should be done to assess the safety of using checkpoint inhibitors after PHP.

## 1. Introduction

Melanoma incidence continues to rise, and unresectable metastatic melanoma is associated with high mortality [1]. Uveal melanoma is a rare subtype of melanoma that progresses to metastatic disease in over 50% of patients [2]. Approximately 20-30% of patients will die of systematic metastases within 5 years of being diagnosed [3]. The development of immune checkpoint inhibitors, particularly those targeting cytotoxic T-lymphocyte-associated antigen 4 (CTLA-4) and programmed cell death-1 (PD-1), has dramatically improved long-term prognosis for patients with advanced cutaneous melanomas [4–7]. However, responses in advanced uveal melanoma have been very limited, so there remains no FDA-approved standard of care for metastatic uveal melanoma [8].

Immune checkpoints mediate immune response and prevent tissue damage [5]. Immune checkpoint receptors, such as PD-1 and CTLA-4, serve as an “off switch” for T-cell functions in tissues, such as destroying other cells [6]. Tumor cells take advantage of this by upregulating ligands for PD-1, thereby blocking the antitumor response. Anti-PD-1 inhibitors block this PD-1 pathway and serve as an “on switch” to allow for the antitumor response to take place and cause tumor regression.

Anti-PD-1 inhibitors, such as nivolumab, serve as an ideal treatment option for uveal melanoma due to their low side effect profile. However, anti-PD-1 inhibitors have been known to induce relatively mild immune-related adverse events (irAEs) such as fatigue, pruritis, and arthritis [9, 10]. Serious adverse events occur in <10% of patients taking nivolumab and include interstitial pneumonitis, hepatitis, hypothyroidism, diarrhea/colitis, adrenal insufficiency, and pituitary insufficiency [9, 11, 12]. The incidence of grade 3 or 4 immune-related hepatitis is only seen in about 1% of patients, while fulminant hepatic failure has only rarely been reported [9, 13].

Chemosaturation with percutaneous hepatic perfusion (PHP) is a therapy that delivers chemotherapy in high doses directly to the liver with the goal of targeting hepatic metastases [14, 15]. One phase III randomized trial in patients primarily with uveal melanoma with liver metastases demonstrated a significant improvement in progression-free survival (PFS) from 1.6 months to 5.4 months when compared to the best available care. Further clinical trials are being done in the United States, but it is currently used as a treatment for uveal melanoma in other countries [16]. Early animal studies have shown high rates of bone marrow depression, but studies have not measured the effect it may have when given with immunotherapy [15].

We present the case of a patient with metastatic uveal melanoma treated with PHP followed by nivolumab who developed fulminant hepatic failure.

## 2. Case Presentation

A 46-year-old man with no significant past medical history presented with blurry vision and was ultimately diagnosed with choroidal melanoma of the right eye. He underwent plaque brachytherapy and was followed with surveillance imaging. Fifteen months after his original diagnosis, MRI abdomen revealed multiple hepatic lesions. A CT-guided liver biopsy of one of the lesions was performed, and pathology confirmed metastatic uveal melanoma. Initial staging showed only liver metastases. The patient travelled to the United Kingdom and received four cycles of chemosaturation with PHP using high-dose melphalan over a 6-month period. Repeat imaging initially showed a partial response to this treatment in the liver. After 6 months, the patient started to complain of rib pain and back pain. A PET/CT scan revealed diffuse bony metastases. He was treated with palliative radiation prior to initiation of nivolumab at 3 mg/kg every two weeks.

**Figure 1:**
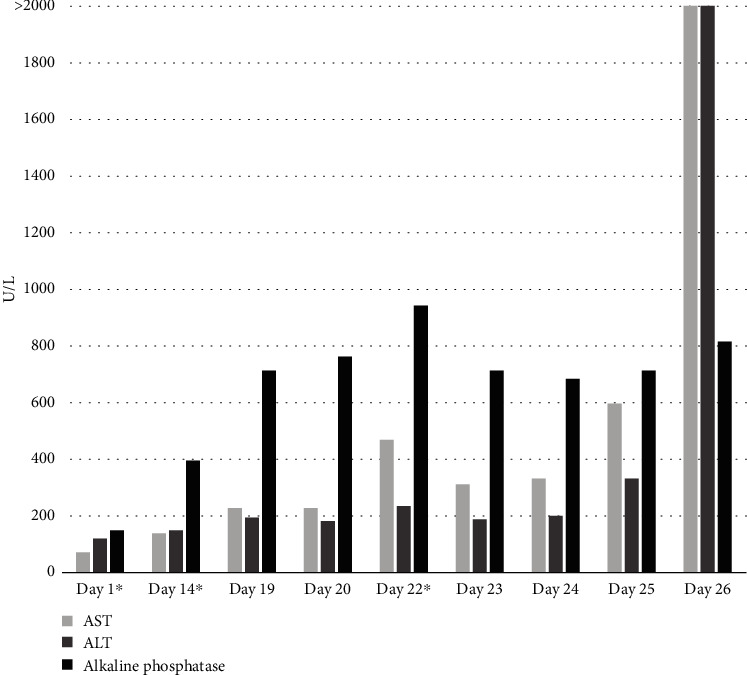
Liver function tests before, during, and after 2 cycles of nivolumab. The dates are numbered starting with the first day of the first cycle of nivolumab. AST: aspartate aminotransferase; ALT: alanine aminotransferase; day 1∗: start of the 1^st^ cycle of nivolumab; day 14∗: start of the 2^nd^ cycle of nivolumab; day 22∗: 1^st^ dose of corticosteroids. The graph shows the rapid increase in liver function tests after the patient began taking nivolumab and the minor response after being treated with corticosteroids. It also depicts the timeline until the patient perspired.

**Figure 2:**
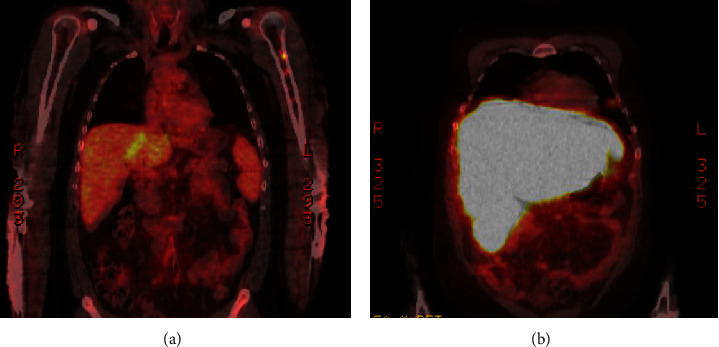
PET/CT scan of the abdomen. (a) One month prior to the first cycle of nivolumab: multiple intrahepatic lesions. (b) Day 22 after the first cycle of nivolumab: significant hepatomegaly due to hepatitis, significant increased metabolic activity of the entire liver, and new abdominal ascites. The PET/CT scan of the patient abdomen depicts the changes in the patient's liver before and after starting nivolumab. The second picture shows the large size of the liver when the patient developed autoimmune hepatitis.

Shortly after the first cycle of nivolumab, he began having mild right upper quadrant pain, low-grade fevers, diaphoresis, and fatigue. Two weeks later, he was given a second dose of nivolumab. Then, one week after that, his right upper quadrant abdominal pain worsened, and he was found to have an aspartate aminotransferase (AST) level of 310 U/L, alanine aminotransferase (ALT) of 187 U/L, alkaline phosphatase of 713 U/L, and total bilirubin of 3.8 mg/dL (Figure 1). Clinical exam revealed marked hepatomegaly. Given his extensive liver metastases, there was an initial question about progression of his disease versus an autoimmune hepatitis, so a PET/CT scan was performed revealing an enlarged liver along with diffusely homogenous uptake in the liver. This suggested a diffuse inflammatory process involving the entire liver rather than progression of metastatic disease which would have shown more heterogeneous FDG avidity corresponding to the sites of tumor activity (Figure 2).

The patient was admitted to the hospital, and further lab workup including anti-nuclear antibodies, anti-smooth muscle antibodies, anti-liver kidney antibodies, anti-mitochondrial antibodies, and IgG levels was all unremarkable. The patient was started on high-dose intravenous Solumedrol and liver function tests (LFTs), and symptoms initially improved during his first few hospital days. However, on the fourth hospital day (day 26 in Figure 1), his condition dramatically declined. His LFTs rose rapidly to the thousands, resulting in fulminant liver failure. He then developed acute renal failure, anion gap metabolic acidosis, and hyperkalemia. The patient was placed on a bicarbonate drip, and efforts were made to reverse his acidosis to stabilize him; however, his condition continued to decline and LFTs continued to worsen (AST: 7,708 U/L, ALT: 3,131 U/L, alkaline phosphatase: 817 U/L, and total bilirubin: 11.9 mg/dL). Mycophenolate mofetil 1,000 mg IV was administered with no improvement. The patient was not a liver transplant candidate because of his metastatic cancer diagnosis. The fulminant hepatic failure was irreversible and led to death secondary to cardiorespiratory arrest.

## 3. Discussion

To our knowledge, this is the first reported case of fulminant hepatic failure secondary to nivolumab in a patient with prior PHP treatment. Immune-related hepatitis from PD-1 inhibitors is usually mild-to-moderate and self-limited, but in less than 1.0% of the patients, the hepatitis is considered severe [17]. This is a result of impaired self-tolerance from the loss of T-cell inhibition and can lead to autoimmune-related inflammation in normal, noncancerous tissues. These adverse events can usually be treated by modulating the immune system with steroids or other immunosuppressing agents, but in some cases, such as this one, it can be fatal.

Mild-to-moderate hepatitis usually occurs between 6 and 14 weeks after starting treatment; however, the patient in our case developed hepatitis in under 4 weeks after starting treatment [18]. One study found that liver toxicity was most commonly seen after 2 to 6 cycles of nivolumab, which aligns with our case [17]. However, there is no established timeline for fulminant liver failure, as it is rare and has only been reported a handful of times in the literature. One case of fulminant hepatic failure due to nivolumab was reported in October 2018 [19]. However, this patient was also taking a CTLA-4 inhibitor, ipilimumab, concurrently. The combination of CTLA-4 and PD-1 blockade is known to cause a higher incidence of hepatitis than single-drug immunotherapy, with the incidence being 6.9% and 1%, respectively [20]. Another patient taking nivolumab after carboplatin and gemcitabine suffered from fulminant hepatic failure [21]. In 2018, a patient with malignant melanoma underwent 17 cycles of nivolumab before developing grade 4 ALT elevation [22]. After steroids, his ALT did not recover to normal range, even after 5 months. Fulminant hepatic failure has also been seen with the PD-1 inhibitor, pembrolizumab [23].

In this case, the patient had been previously exposed to PHP to treat liver metastases from uveal melanoma. As discussed above, in phase 3 randomized controlled trials (RCTs), PHP has been shown to be efficacious when compared to chemotherapy, but it can also lead to adverse events. One RCT found the rate of grade 3 or 4 hepatic dysfunction, diagnosed by hyperbilirubinemia, to be 14.3% [16]. There were also 5.7% of patients in the RCT that discontinued treatment because of hepatic toxicity, diagnosed by hyperbilirubinemia (5.7%), increase in ALT (2.9%), and increase in AST (2.9%) [16]. One retrospective review of 14 patients who underwent PHP found 1 patient who had hepatic failure that resulted in death [24].

There is limited knowledge regarding concurrent immunotherapy and PHP treatment, as patients undergoing immunotherapy have been excluded from PHP RCTs [16]. However, there is a case report of a man who began immunotherapy with ipilimumab 10 weeks after PHP, resulting in gallbladder toxicity [25]. In our case, the patient had normal LFTs after receiving PHP and before starting nivolumab. It was not until he received two doses of nivolumab that his LFTs began to quickly escalate. Given his prior PHP treatments, he may have been at increased risk for immune-related hepatitis from nivolumab.

## 4. Conclusion

In conclusion, since fulminant hepatic failure from nivolumab alone is rare and PHP is also known to cause hepatic toxicity and hepatic failure, we believe that PHP preceding nivolumab contributed to fulminant hepatic failure. Healthcare providers should use caution in starting immunotherapy for patients who have previously received PHP therapy. Careful monitoring of liver function tests and early intervention for hepatic deterioration should be implemented in all patients receiving immunotherapy.

## Data Availability

The datasets generated and/or analyzed during the current study are not publicly available due to individual privacy but are available from the corresponding author on reasonable request.

## Abbreviations

ALT: Alanine aminotransferase
AST: Aspartate aminotransferase
CTLA-4: Cytotoxic T-lymphocyte-associated protein 4
LFT: Liver function test
PD-1: Programmed death 1 receptor
PD-L1: Programmed death 1 receptor ligand
PHP: Percutaneous hepatic perfusion
RCT: Randomized controlled trial.
